# The Oath of Hippocrates*From *Bulletin of the Johns Hopkins Hospital*.

**Published:** 1892-06

**Authors:** 


### THE OATH OF HIPPOCRATES.*

The oath of Hippocrates forms the basis of higher medical ethics in human medicine, and from a broad standpoint, is not out of place, and may furnish some useful hints to the veterinarian. The following paper by F. R. Smith, Assistant Resident Physician of Johns Hopkins Hospital, is at least interesting :—

"THE OATH. *῞Ορκος*.—I swear by Apollo the physician, and Æsculapius and Health and All-Heal and all the gods and goddesses, that according to my ability and judgment I will keep this oath and stipulation—to reckon him who taught me this art equally dear as my parents, to share my substance with him, and relieve his necessities if required ; to look upon his offspring on the same footing as my own brothers, and to teach them this art if they shall wish to learn it, without fee or stipulation ; and by precept, lecture, and every other mode of instruction I will impart a knowledge of the art to my own sons and those of my teachers, and to disciples bound by a stipulation and oath according to the laws of medicine, but to none others. I will follow that system of regimen which, according to my ability, I consider for the benefit of my patients, and abstain from whatever is deleterious and mischievous. I will give no deadly medicine to any one if asked, nor suggest any such counsel ; and in like manner I will not give to a woman a pessary to produce abortion. With purity and holiness I will pass my life and practice my art. I will not cut persons laboring under the stone, but will leave this to be done by men who are practitioners of this work. Into whatever houses I enter, I will go into them for the benefit of the sick, and will abstain from every voluntary act of mischief and corruption ; and further, from the seduction of females or males, of freemen or slaves. Whatever in connection with my professional practice, or not in connection with it, I see or hear, in the life of men, I will not divulge, as reckoning that all such should be kept secret. While I continue to keep this oath unviolated, may it be granted to me to enjoy life and the practice of the art respected by all men, in all times ! But should I trespass and violate this oath, may the reverse be my lot ! "

After all the controversy that has raged with respect to the genuineness of this document, the weight of evidence seems to favor the opinion that the "oath" was written either by Hippocrates or one or other of his immediate disciples, despite the important fact that

* From *Bulletin of the Johns Hopkins Hospital.*

Galen does not include it in his list. There is nothing in the internal evidence which would give us a definite date. The appeal in the opening to special gods as patrons of medicine would show that it did not come from prehistoric times, in which all the gods were equally powerful in the healing art, while again, the assertion of Sprengel that this invocation shows that the work issued from the Alexandrian school cannot be held to be more than a supposition. Beyond this, as we read it we are convinced that it is the work of doctors and of gentlemen—of men educated, as far the times would allow, in the mysteries of the healing art, but who at the same time understood the proper position of the doctor in society, and who thought it right to insist upon the responsibility of all who dared to undertake such duties, and the necessity of not disgracing one of the noblest professions.

In most places the text speaks for itself, and we shall confine ourselves more especially to two passages which have always presented some difficulty, and put forward some of the ideas that have been expressed on these points. The first of these is the one where the methods of teaching are mentioned; the second, where the prohibition of lithotomy is dealt with. But first we would touch lightly on a few less important points.

*The opening invocation.*—In very ancient times healing belonged to all the gods alike; then certain of the gods were chosen, and represented as taking special interest in this art. With the natural tendency to seek to propitiate what men fear, seen so clearly in the names given to terrible persons and objects in ancient as well as in medieval times—as the "inhospitable" sea came to be called the Euxine or hospitable sea, the Furies the kind maidens, as the dreaded fays and sprites were mentioned with bated breath as the "good people," so Apollo the destroyer became Apollo the averter of evils and "he who distributes to much-suffering mortals the medicines of the Asclepiads."

Whatever may have been his faults, Apollo could not have been idle; for besides the art of medicine, he had also under his special care prophecy, education and the art of shooting with the bow. In many a country place his representative may still be found in the person of the hard-worked country doctor. He is the weather prophet, he assists at the counsels of the school board; on medicine his decision is final, and often, perhaps, when his presence is badly needed he may be found to have gone hunting.

*Without fee or stipulation.*—The teaching descended from father to son, and at first no others were taken into this close brotherhood. But later, certain others were admitted, and certainly from these pupils it was lawful to take pay. The young man mentioned by Plato as anxious to study medicine went prepared to pay for his tuition, and Galen refers to the introduction of strangers into the profession when he says that books on anatomy were not necessary as long as the teaching went down from father to son.

*Abortion.*—The stand taken by Hippocrates on this point differs widely from that of some of his contemporaries. Even Aristotle does not object to the operation if it be done before the time of quickening; and again Hippocrates represents the best opinions of the present day, though the idea of Aristotle seems to have had some effect on the popular mind which has not yet died out.

*Giving drugs for the purpose of poisoning.*—A word on this subject was surely necessary. We need not expatiate on the prevalence of poisoning everywhere all over the world, which seems to have culminated in the state of things described by Juvenal, who speaks of it as an everyday occurrence. Writing to a friend and inviting him to dinner, the satirist, after giving the *menu*, adds, with grim humor, "and mushrooms I will give thee; not of that kind which the wife of Claudius gave him for supper, after which he ate no more." He also mentions a lady who not only had poisoned several husbands, but influenced the other women "so that they dared to have their husbands, too, carried out to burials with faces black" (from poison). But passing from this, the passage leads us to enquire: "Did the physician compound his own drugs?" We must suppose so. It is true that there were φαρμακοπωλαι or sellers of drugs, but these were men who dealt in poisons, madstones, charms, secret remedies, but they do not seem to have dispensed prescriptions. If Eudemus was a true representative of the class, and Aristophanes is to be believed, they were charlatans, such as would swallow poisons publicly to show the efficiency of of their antidotes.

Later on we have, however, the πημενταριοσ, who, Olympiodorus tells us, made up the prescriptions of the physicians. Thus we have a system of things that may be seen in Germany to-day. The doctors prescribe, the prescriptions are made up in the licensed drug stores, and, in addition, we have the tradesmen, more humble but more honest than the old pharmacopolæ, the sellers of herbs and drugs, such as are more usually bought for household or trade purposes. Pliny says the practice of writing prescriptions was carried so far that the doctors prescribed drugs of which they knew absolutely nothing.

*I will not cut persons laboring under the stone* (ου τεμέω δε ουδὲ μην λιθιωντασ). If this is really what the passage means, we are still at a loss to understand why Hippocrates should forbid his disciples to perform this operation. It was not because surgery was beneath the dignity of members of this school, for Cicero says, "Think ye that in the time of Hippocrates of Cos that there were special physicians, some for internal sickness, others for wounds, others for the eyes?"

Herodotus, indeed, in his book on Egypt, tells that there were specialists for diseases of every part of the body; but did not Hippocrates himself or his followers write on all kinds of diseases and on surgery also, and how are we to explain his reasons for prohibiting only this operation for stone? Were there, then, specialists for this operation alone? We may infer that it was no new or strange operation, for Celsus tells us of one Ammonius of Alexandria who invented an instrument for crushing a calculus too large to be taken away whole through the perineal wound, and in mentioning this circumstance he seems to assume that the operation was well known.

Again, Hippocrates himself explains the use of the sound in demonstrating the existence of a calculus; why then should he stop short here and refuse to relieve the patient himself? The assumption that there were specialists for this operation and for none other, we have no means of proving, neither can the date of the invention of the operation be fixed from any records that have come down to us.

Must we then, refusing to accept the simplest interpretation of the Greek, here look for a hidden meaning and see in these words the prohibition of castration? We cannot indeed accept the translation of René Moreau, "I will not cut those who have not stones;" but it would make things rather easier if we could be convinced that Hippocrates was condemning here a disgraceful operation, and not one that has proved of such great benefit to many sufferers. In our days castration is employed in order to supply ennuchs for the Eastern harems, and not so long ago it was practised in Italy on the "Castrati," whose beautiful soprano voices have doubtless pleased the ears of many of us in the churches. But, if not in the time of Hippocrates, at least in the time of the empire at Rome, castration had for its object the gratification of much worse passions. The Roman ladies, in order to avoid the trouble of conception, were wont to have favorite slaves castrated that there might be no drawback to the indulgence of their lusts. Heliodorus is mentioned as a type of the practitioner to whom they resorted. So flagrant became the custom that edicts against it were promulgated by more than one emperor. Later again, a physician, one Paul of Ægina, gives two methods of performing the operation. He regrets the necessity of it, but naïvely remarks that some of the rich patrons might possibly demand it and the physician be unable to resist the pressure brought to bear upon him. We might say that τεμέω is not exactly the word we might have expected in the sense of "castrate"—ἐκτέμνω or αποτέμνω· would probably have been more usual.*

*Further history.*—Of the further history of the oath we can say but little. We give here a mutilated form, which dates from the early centuries of the Christian era.

"Before the great God himself I swear that I will destroy no man wilfully—no man, whether he be a fellow-countryman or stranger, by any homicidal practice; that none shall induce me by bribes to commit a horrible crime by giving to any man noxious drugs capable of causing mortal injury; that even out of friendship I will not give them. But I raise to heaven my hands unstained and keep my thoughts unsullied by crime. I will do everything I can for the sick, and to all I will try to produce health."

Of this oath a critic satirically remarks, "All this could be found in the penal statutes."

As our oath is in some measure an agreement between pupil and master, it may not be out of place to insert here extracts from the form of "indentures" used in England nearly up to the present time.

"A. doth apprentice himself to B. to learn his art, and with him, after the manner of an apprentice, to serve from —— to —— unto the full end and term of five years from thence next following, etc. During which time the said apprentice his master faithfully shall serve, his secrets keep, his lawful commands everywhere gladly do. He shall do no damage to his said master, nor see to be done of others, but to his power shall tell, or forthwith give warning to his said master of the same. He shall not waste the goods of his said master, nor lend them unlawfully to any. He shall not commit fornication nor contract matrimony within the said term, shall not play at cards or dice tables or any other unlawful games whereby his said master may have any loss with his own goods or those of others; during the said term, without license of his said master he

* Possibly we find here ethics for certain veterinary operations.—Ed.

shall not buy nor sell, he shall not haunt taverns or play-houses or absent himself from his said master's service day or night unlawfully, but in all things as a faithful apprentice he shall behave himself towards his said master and all his during the said term; and the said master his said apprentice in the art of medicine and surgery which he useth, by the best means that he can, shall teach and instruct or cause to be taught and instructed, finding unto the said apprentice sufficient meat, drink, lodging and all other necessaries during the said term," etc.

A modified form of the oath is still in use at some places, in McGill University in Canada, and perhaps also in Montpellier and other medical schools. For many years the surgeons at St. Thomas Hospital in London had to subscribe to the clause which forbids the cutting for stone.

Modern medical ethics, as they were put forward to us as students, looked at a doctor's position from two very different standpoints: (1), the duty of the physician to his patient and to his colleagues; (2), the duty of the patient to his physician.

If the formulators of our oath have made a serious omission, it is one not difficult to supply. There is no mention of high fees, no promise of making fortunes. We need not then assume that the laborer is not worthy of his hire, but the keynote of the whole appears to be "duty," and Hippocrates would have us learn that in the consciousness of duty done, and in nothing else, may his true followers hope for their reward.

